# Eosinophilic Esophagitis: Mechanisms of Disease and Approach to Treatment

**DOI:** 10.1007/s11882-026-01253-w

**Published:** 2026-03-12

**Authors:** Ravi Gautam, Melanie A. Ruffner

**Affiliations:** 1 Division of Allergy and Immunology, Children’s Hospital of Philadelphia, Philadelphia, PA USA; 2 Department of Pediatrics, University of Pennsylvania Perelman School of Medicine, Philadelphia, PA USA

**Keywords:** Eosinophilic esophagitis (EoE), Pathophysiology, Type 2 inflammation, Epithelial barrier dysfunction, Eosinophils, Mast cells, Alarmins (TSLP, IL-25, IL-33), Fibrostenosis, Biologic therapies, Immune modulation

## Abstract

**Purpose of Review:**

This review examines the underlying mechanisms of disease pathogenesis in eosinophilic esophagitis with the aim of identifying how current and emerging therapies target specific pathologic pathways.

**Recent Findings:**

Over the past three decades, the incidence and prevalence of EoE have risen significantly and it is now a leading cause of dysphagia and food impaction in both children and adults. Recent studies highlight how genetic predisposition, environmental exposures, epithelial barrier dysfunction, and abnormal type 2 immune responses interact to contribute to EoE pathogenesis. Key mechanisms include the release of epithelial alarmins, elevated type 2 cytokine signaling, and the recruitment of immune effector cells, all of which lead to chronic inflammation, tissue remodeling, and fibrostenosis. Current treatments include dietary management, proton pump inhibitors, swallowed corticosteroids, and dupilumab, the first approved biologic. New biologics and agents targeting the epithelial barrier, IL-13, TSLP, and related pathways offer hope for more personalized, mechanism-based therapies.

**Summary:**

Current therapies for EoE aim to reduce inflammation and prevent fibrostenotic complications, but challenges such as treatment nonresponse and long-term disease progression still exist. Mechanism-based therapies, particularly biologics targeting type 2 inflammatory pathways and barrier dysfunction, represent a promising frontier for more personalized treatment strategies. Understanding the interplay of immune signaling and epithelial dysfunction can inform the development of next-generation therapies with the potential to transform outcomes in EoE.

## Introduction

Eosinophilic esophagitis (EoE) is a chronic, immune-mediated condition marked by esophageal dysfunction and inflammation caused by an abnormal type 2 (Th2) immune response and dense eosinophilic infiltration into the esophageal mucosa [1]. Since it was identified as a distinct disorder in the 1990 s, eosinophilic esophagitis (EoE) has become increasingly diagnosed and is now a major cause of upper gastrointestinal problems. EoE is the second most common cause of chronic esophagitis after gastroesophageal reflux disease (GERD) and is the leading cause of dysphagia and food impaction in adolescents and adults [2].

Epidemiological data highlight the increasing burden of EoE. A global meta-analysis of studies published between 1976 and 2022 reported a pooled incidence of 5.31 cases and a prevalence of 40.04 cases per 100,000 inhabitants-year [3]. Significantly higher rates were observed in high-income countries, especially in North America and Europe, compared to low- or middle-income nations. In the United States, the prevalence of EoE is estimated at 1 in 700 individuals, with associated annual healthcare costs of $1.3 billion annually [4].

The diagnosis of EoE depends on both clinical symptoms and histologic criteria. EoE can affect people of all ages, with its clinical manifestations varying according to age. In children, EoE usually presents with symptoms like vomiting, abdominal pain, feeding difficulties, and inadequate weight gain. Conversely, adolescents and adults are more likely to exhibit progressive esophageal dysfunction caused by fibrostenosis, which appears as dysphagia and food impaction [5]. Patients presenting with symptoms suggestive of EoE should undergo endoscopy accompanied by biopsy to confirm esophageal eosinophilia. To meet the diagnostic criteria for this condition, there must be at least 15 intraepithelial eosinophils per high-power field (HPF, or roughly 60 eosinophils/mm²) seen in biopsy samples [6].

Understanding of EoE pathophysiology has progressed significantly, and ongoing research into the disease mechanisms continues to reveal new pathways that could be important therapeutic targets. Current evidence shows that EoE is a complex disorder and underscores the central role of abnormal type 2-mediated inflammation, epithelial barrier disruption, eosinophilic infiltration, and tissue remodeling, all of which contribute to its clinical and histopathological features [7, 8]. Standard treatment for EoE generally involves three main strategies: dietary changes, medication, and esophageal dilation when strictures are present. Effective medication options include proton pump inhibitors (PPIs), swallowed corticosteroids, and dupilumab [9]. Here, we will review current knowledge of EoE pathophysiology and assess both established and emerging treatments.

### Pathophysiology of EoE

#### Genetic and Environmental Predisposition

EoE is recognized as a complex condition influenced by the interplay between genetic and environmental factors. Early studies on the familial inheritance of EoE estimated that 2.4% of siblings of probands also had EoE, indicating a 40-fold increased risk compared to the general population. The incidence of EoE was notably higher in monozygotic twins (41%), but the increased risk in dizygotic twins (21%) compared to sibling pairs highlights the significant role of environmental factors, especially during early life, in the development of EoE [10].

Environmental factors, such as maternal fever, preterm labor, cesarean delivery, and a subset of neonatal exposures (including antibiotics, acid suppressants, formula feeding, cold climates, and indoor pollutants), have been linked to an increased risk of developing EoE [11–13]. Dysbiosis and limited microbial exposure during early childhood have been hypothesized to play a critical mechanistic role in EoE development given the nature of these specific risks. Furthermore, patients with active EoE exhibit specific changes in the esophageal microbiota, including increased levels of Haemophilus and Aggregatibacter species, and decreased levels of Firmicutes [14]. Notably, exposure to Helicobacter pylori has been significantly associated with a reduced risk of developing EoE [15].

Genetic studies have uncovered the multifactorial origins of EoE and identified candidate molecules that affect epithelial barrier function, immune signaling, and tissue remodeling. Genome-wide association studies (GWAS) have identified multiple risk loci associated with compromised epithelial barrier, including the 2p23.1 locus that contains *CAPN14*,* which* encodes Calpain 14 [16]. Calpain 14 is an esophageal-specific protease that is upregulated by T2 cytokine/STAT6 signaling and damages the epithelial barrier by degrading desmoglein 1 [17, 18]. Rare variants in *DSG1* and *DSP* have been associated with familial EoE, emphasizing the role of desmosomal disruption in the disease’s pathophysiology [19]. Loss-of-function (LOF) variants of filaggrin are more frequently found in individuals with EoE, and esophageal epithelial filaggrin levels are also reduced in the presence of high T2 cytokine levels, leading to an acquired decrease of filaggrin in EoE patients regardless of mutations in the gene [20].

Additional risk loci emphasize the role of specific immune pathways in the inflammation and tissue remodeling seen in EoE. Several studies report variants at 5q22.1, which includes the *TSLP* and *WDR36* loci [21]. Thymic stromal lymphopoietin (TSLP) is an epithelium-derived alarmin cytokine that promotes allergic Th2 immune responses and directly influences various allergic effector cells, including eosinophils, mast cells, and basophils [22, 23]. TSLP is overexpressed in EoE, while WDR36 remains unchanged, confirming TSLP as the primary gene affected at this locus [21]. Moreover, individuals with risk variants at both the *TSLP* and *IL4* loci have a 3.7-fold increased odds of developing EoE, compared to odds ratios of 1.3 and 1.6 when carrying either *TSLP* or *IL4* alone [24]. Similarly, multiple studies identify variants at the 11q13.5 locus, which encodes *LRRC32* and *C11orf30/EMSY*. *LRRC32* encodes a Transforming growth factor-beta (TGF-β)-binding protein involved in regulatory T-cell-mediated suppression of colitis and is upregulated by IL-13. EMSY is linked to epithelial cell differentiation. Both genes are expressed in esophageal epithelial cells. In addition to these loci, several other genes with critical immune functions have been linked to EoE risk, including *CCL26* (which encodes eotaxin-3, a chemokine involved in eosinophil recruitment), *STAT6*, and *ITIH5* [25–27].

EoE also occurs more frequently in several Mendelian syndromes. These include Loeys-Dietz syndrome, which involves defects in the TGF-β pathway, and connective tissue disorders such as Marfan syndrome and Ehlers–Danlos syndromes [28]. Disorders like Netherton syndrome (*SPINK5* LOF) and Severe Atopy with Metabolic Wasting (*DSP* LOF) highlight the role of the epithelial barrier in EoE [29, 30]. Additionally, *STAT6* gain-of-function variants have been linked to severe atopic dermatitis, eosinophilic esophagitis, and other atopic conditions [31]. Autosomal dominant hyper-IgE syndrome, caused by *STAT3* gene mutations, is also associated with a high prevalence of gastrointestinal manifestations, including eosinophilic gastrointestinal disorders [32, 33]. Patients with germline mutations in the tumor suppressor *PTEN* develop noncancerous hamartomas and are at increased risk of malignancies over time, with studies reporting higher rates of eosinophilic gastrointestinal disorders [34].

#### Epithelial Barrier Dysfunction

Patients diagnosed with eosinophilic esophagitis (EoE) display various morphological and functional abnormalities in the esophageal epithelium. Notable features include dilated intercellular spaces (DIS) and basal zone hyperplasia (BZH), which result from widened spaces between the suprabasal layers, dysregulation of epithelial differentiation, and expansion of the undifferentiated basal cell layer [35, 36]. At the molecular level, approximately 40% of esophageal-specific genes involved in processes like keratinization and cellular differentiation are downregulated [37]. Moreover, there is a loss of essential proteins critical for maintaining mucosal integrity and facilitating cell-to-cell junction formation, including filaggrin [38, 39], desmoglein-1, claudins, occludin, E-cadherin, involucrin [37], and zonula occludens-3 [39].

Impaired esophageal epithelial barrier function is a key factor in EoE pathophysiology because compromised barriers allow food or environmental antigens to penetrate and worsen inflammation in EoE [40, 41]. This triggers the release of epithelial alarmins, including TSLP, interleukin-25 (IL-25), and IL-33 [42], which promote type 2 inflammation through ILC2 activation and Th2 polarization, leading to the production of IL-4, IL-5, and IL-13 [43]. IL-4 and IL-13 signaling is mediated by JAK/STAT pathways, resulting in multiple effects within the esophageal tissue. These cytokines decrease the expression of epithelial differentiation complex molecules, such as filaggrin, LOR, and involucrin, via STAT6- and STAT3-dependent mechanisms [44]. Serine peptidase inhibitor kazal-type 7 (SPINK7) inhibits proteolytic activity in the esophagus and is downregulated by type-2 cytokines, which increases proteolytic activity and reduces barrier function [45].

Additional cytokines in the inflammatory environment contribute to epithelial dysfunction and tissue remodeling in EoE. IL-20 subfamily cytokines (IL-19, IL-20, IL-24) are elevated in EoE and directly inhibit genes that regulate epithelial differentiation, desmosomes, and tight junction proteins [46]. TGF-β1 is increased in EoE, reducing epithelial claudin-7 and weakening junctional integrity [47]. Similarly, IL-9 has been shown to downregulate E-Cadherin [48]. Oncostatin M (OSM), a member of the IL-6 family, is elevated in EoE and decreases the expression of desmoglein 1, filaggrin, involucrin, and increases barrier permeability [49, 50].

Lastly, products produced by eosinophils and mast cells during activation and degranulation contribute to epithelial barrier function. Eosinophil granule proteins, such as major basic protein (MBP), can damage the esophageal epithelium and potentially impair barrier function [51]. Mast cells are increased in the mucosa of patients with EoE, and their presence has been associated with increased esophageal symptoms, including pain [52–54]. Mast cell-derived cytokines, including granulocyte-macrophage colony-stimulating factor and oncostatin M, have been linked to decreased expression of filaggrin and desmoglein 1 [50].

#### Cellular and Immune Components in the Inflammatory Pathology of EoE

EoE is a chronic, antigen-driven inflammatory disease characterized by a type 2 immune response initiated at the epithelial barrier. Damaged or allergen-exposed epithelial cells release alarmins, such as TSLP, IL-33, and IL-25 (see Table 1: *Therapeutically relevant alarmins and chemokines in EoE*). Activation of innate sensing mechanisms, like the RIPK1-Caspase-8 ripoptosome complex, have been shown to activate IL-33 secretion from epithelial cells [55]. Transgenic mice overexpressing IL-33 in the esophageal epithelium spontaneously develop an EoE-like phenotype, suggesting that IL-33 plays a key role in initiating EoE [56]. TSLP is increased in the esophageal tissue of EoE patients, and TSLP receptor-deficient mice are protected from experimentally induced EoE, indicating that TSLP also plays an essential role in promoting inflammation in EoE [57, 58]. Epithelial alarmin and chemokine secretion recruit dendritic cells, basophils, iNKT cells, and ILC2s, thereby establishing a pro-Th2 microenvironment [59].

**Table 1:** Therapeutically relevant epithelial alarmins and chemokines in EoE

Category	Mediator	Major Functions in EoE	Therapeutic Targeting (approved or in clinical trials)
Epithelial alarmins	TSLP	Activates dendritic cells, basophils, ILC2s; initiates Th2 inflammation	Anti-TSLP (Tezepelumab) approved for asthma; in phase 3 trials for EoE
	IL-33	Promotes Th2 inflammation via ILC2s, mast cells; drives epithelial inflammation	Anti–IL-33 (etokimab, astegolimab) trials in asthma/atopy, early studies in EoE
	IL-25	Enhances ILC2 activity; amplifies Th2 cytokine production	No EoE-specific drug trials yet
Epithelial-derived chemokines	CCL26 (Eotaxin-3)	Recruits eosinophils via CCR3	CCR3 antagonists under study in allergy/asthma; not yet in EoE
	CCL11 (Eotaxin − 2)	Eosinophil chemotaxis via CCR3; less abundant than CCL26	Indirectly targeted by IL-4/IL-13 blockade
	CCL24 (Eotaxin-1)	Eosinophil chemotaxis via CCR3, less abundant than CCL26	Indirectly targeted by IL-4/IL-13 blockade
	CCL17 (TARC)	Recruits Th2 cells via CCR4	Anti-CCR4 mAb (mogamulizumab) approved for oncologic indications; not tested in EoE
	CCL22 (MDC)	Recruits Th2 cells via CCR4	Same as above (anti-CCR4, not tested in EoE)
	CXCL10	Recruits macrophages, DC, and lymphocytes via CXCR3	Anti-CXCL10 therapies in clinical trial for autoimmune disease; not studied in EoE

Th2 cells are polarized and activated by alarmins, and CD4 + pathologic effector Th2 (peTh2) cells are significantly enriched in EoE [60–62]. These cells express the homing receptor GPR15 and produce high levels of hallmark cytokines IL-4, IL-5, and IL-13 [63]. These cytokines, in turn, promote eosinophil survival and recruitment, enhance Th2 responses, and remodel esophageal stromal tissue. Other T-cell populations are also increased in EoE: (see Table 2: *Cytokines as current and potential therapeutic targets in EoE*). There are significantly higher numbers of Th17 and CD8 + T cells producing TNF-α and interferon (IFN)-γ observed in active EoE biopsies [61, 64]. T cells have also been shown to produce the TNF-family cytokine LIGHT (TNFSF14), which activates esophageal fibroblasts and promotes a pro-inflammatory state [65].

**Table 2:** Cytokines as current and potential therapeutic targets in EoE

Category	Mediator	Major Functions in EoE	Therapeutic Targeting
Type 2 cytokine signaling	IL-4	Promotes Th2 differentiation; eosinophil survival	anti–IL-4Rα (dupilumab) blocks IL-4/IL-13; FDA-approved for EoE in ≥ 1 yrs and ≥ 15 kg)
	IL-13	Induces CCL26, barrier dysfunction (CAPN14, DSG1 downregulation), remodeling	Dupilumab (as above) Anti–IL-13 agents (cendakimab, lebrikizumab, tralokinumab — mixed/ongoing EoE trials)
	IL-5	Drives eosinophil proliferation, survival, chemotaxis	Anti–IL-5 (mepolizumab, reslizumab), anti–IL-5R (benralizumab) failed to meet primary endpoints in EoE
Other cytokine signaling	IL-9	Enhances mast cell survival, ILC2 expansion, barrier dysfunction	Not studied in EoE, Anti-IL-9 (enokizumab) failed to show benefit in Phase II asthma trial
	IL-20 family (IL-19/20/24)	Elevated in EoE, promote epithelial dysfunction	Not studied in EoE, blocking antibodies in study for other indications
	IFN-γ	Contributes to epithelial barrier loss; produced by Th1/CD8 T cells	JAK inhibitors (baricitinib, upadacitinib — in related atopic diseases; potential relevance in EoE)
	TNF-α	Promotes EMT, fibrosis, eotaxin-3 synergy	Anti–TNF agents (infliximab, adalimumab) ineffective in small EoE studies
	OSM (Oncostatin M)	IL-6 family cytokine, disrupts barrier proteins (DSG1, FLG, IVL)	Not studied in EoE, blocking antibodies in study for other indications

Eosinophils are the key diagnostic marker of EoE, recruited to the mucosa by eosinophil chemotactic factors (see Table 3: *Effector cell chemotaxis and effector function as a potential therapeutic target in EoE)*. Eotaxin-3 (CCL26) is overexpressed, promoting chemotaxis through the CCR3 receptor [66]. Activated eosinophils degranulate, releasing proteins like MBP, ECP, EDN, and EPO, causing cytotoxicity and inflammation [67]. They also secrete cytokines (IL-2, IL-4, IL-6, IL-10, IL-12, IL-13, GM-CSF, and TNF-α), present antigens via MHC II, and form eosinophil extracellular traps (EETs), thereby contributing to microbial defense and immune modulation [68].

**Table 3:** Effector cell chemotaxis and function as a potential therapeutic target in EoE

Category	Mediator	Major Functions in EoE	Therapeutic Targeting
Adhesion molecules	Siglec-8	Selectively expressed on eosinophils, mast cells, basophils; ligation induces eosinophil apoptosis and inhibits mast cell degranulation	Phase 2/3 trial of anti-Siglec-8 (lirentelimab) met the histologic co-primary endpoint but did not meet the co-primary endpoint of symptom reduction
	CRTH2 (DP2 receptor)	Prostaglandin D₂ receptor on Th2 cells, ILC2s, eosinophils, basophils; mediates chemotaxis and activation	CRTH2 antagonists (e.g., fevipiprant) trialed in asthma; no published EoE data
	α4β7 integrin (LPAM-1)	Mediates lymphocyte homing to gut mucosa; possibly involved in esophageal T-cell trafficking	Anti-α4β7 mAb **(**vedolizumab**)** approved for treatment of IBD; limited EoE case reports but has not been studied
Mast cell function	IgE/FcεRI (High-affinity IgE receptor)	Cross-linking by allergen-specific IgE triggers mast cell degranulation; increased mast cells observed in EoE	anti-IgE mAbs (omalizumab, ligelizumab) minimal benefit in EoE
	KIT (CD117)	Receptor tyrosine kinase critical for mast cell survival and activation	KIT inhibitors (imatinib, masitinib) have not been studied in EoE
	Cysteinyl leukotrienes	Produced by mast cells and eosinophils	Montelukast was not effective at inducing remission in EoE studies

Mast cells are increased in the esophageal mucosa in EoE and can degranulate to release tryptase, chymase, and Cathepsin-G, which are serine proteases and carboxypeptidase A3. These preformed components can directly cause tissue damage. Mast cell degranulation also releases bioactive small molecules such as histamine, heparin, and serotonin that have feed-forward effects on immune cells within the inflammatory environment. Chronic mast cell activation leads to the production of late-phase mediators, including prostaglandins, leukotrienes, and cytokines such as IL-13. These factors promote eosinophil recruitment, barrier dysfunction, vascular permeability, smooth muscle contraction, and fibrosis.

#### Key Inflammatory Mediators

Among cytokines, IL-13 is a key mediator of EoE, exerting multiple effects in this condition. It is highly upregulated in human esophageal tissue during EoE, and in vitro IL-13 treatment reproduces many of the characteristic epithelial gene expression changes seen in EoE biopsy specimens [20, 69]. IL-13 promotes eosinophil recruitment by increasing Eotaxin-3 (CCL26) levels and inducing endothelial VCAM-1 and ICAM-1, thereby aiding leukocyte infiltration into inflamed esophageal tissue [70, 71]. IL-13 also plays a vital role in tissue remodeling by encouraging epithelial hyperplasia, collagen buildup, and angiogenesis [72]. Although less abundant than IL-13, IL-4 is elevated in EoE and works with IL-13 to promote a T2-high environment, enhance eosinophil recruitment, and contribute to esophageal remodeling [73].

IL-5 is markedly increased in esophageal biopsies from patients with active EoE, with higher IL-5 mRNA and protein levels that closely correlate with tissue eosinophil density and disease activity [74]. IL-5 is secreted by Th2 cells, mast cells, and eosinophils, promoting eosinophil growth, survival, and chemotaxis. In mouse models, overexpression of IL-5 in the esophageal mucosa or intratracheal administration of IL-5 can induce esophageal eosinophilia similar to human disease. Additionally, blocking IL-5 or deleting its expression through gene knockout nearly eliminates the disease in the murine IL-13-induced model of EoE, revealing its central role as a mediator of eosinophil chemotaxis [75].

Eotaxins are chemotactic cytokines mainly produced by epithelial cells in response to IL-4 or IL-13 stimulation. They bind specifically to the CCR3 receptor, which is primarily expressed on eosinophils and mast cells, mediating their targeted recruitment in EoE [76, 77]. The eotaxin family includes three members: eotaxin-1, eotaxin-2, and eotaxin-3, with eotaxin-3 being the most highly expressed chemokine in EoE patients [27, 78]. A single nucleotide polymorphism (SNP) on chromosome 7q11 at the *CCL26* locus has been linked to higher eotaxin-3 levels and an increased risk of EoE, likely by enhancing mRNA stability [27, 79, 80]. In a mouse model of EoE, genetic deletion of CCR3 effectively blocked eosinophil infiltration and prevented the disease from developing, highlighting the key role of the eotaxin-3/CCR3 axis in EoE pathogenesis [27].

Periostin is significantly upregulated in patients with EoE and functions as a cell adhesion molecule that regulates extracellular matrix deposition [81]. Both IL-13 and TGF-β induce periostin production from esophageal fibroblasts as well as epithelial cells, and periostin has been shown to facilitate eosinophil adhesion and recruitment into the esophagus [82].

Other mediators broaden the inflammatory spectrum and contribute to the complex inflammatory environment seen in EoE. IL-20 cytokine family members (IL-19, IL-20, IL-24) are elevated in EoE and have been shown to disrupt epithelial barrier function through the mitogen-activated protein kinases (MAPK)/extracellular-signal-regulated kinase (ERK)1/2 pathway. TGF-β1 levels are increased in esophageal biopsies of EoE patients; it is produced by locally activated eosinophils and mast cells and is the most extensively analyzed cytokine in EoE-associated fibrous remodeling [83, 84]. Interferon gamma (IFN-γ) producing CD8 + T cells, and interferon response signature genes are elevated in EoE biopsy tissue. IFN-γ reduces epithelial barrier function of human esophageal epithelial cells, indicating a potential synergistic role with T2-cytokines in EoE [85].

The specific role of some immunologic mediators in fibrosis has been examined. TGF-β promotes esophageal remodeling by activating fibroblasts and stimulating the secretion of ECM proteins, thereby promoting smooth muscle proliferation, hyperplasia, and contractility. It also drives epithelial-mesenchymal transition (EMT), during which epithelial cells acquire myofibroblast features and lose certain epithelial characteristics [41].

TNFα is upregulated in EoE and is highly expressed by esophageal epithelial cells in patients with active disease [86]. TNFα acts in conjunction with IL-4 and IL-13 to induce eotaxin-3, and is thought to promote epithelial-to-mesenchymal transition and esophageal fibrosis [87, 88]. Both TRAIL (TNFSF10) and its downstream effector MID-1 are significantly upregulated in esophageal biopsies. In murine models of allergen-induced EoE, TRAIL knockdown led to reduced inflammation and decreased markers of fibrosis [89].

Finally, although IgE and IgG4 are often elevated in EoE patients, their roles seem to be indirect [41, 90]. IgE may facilitate mast cell activation in some patients, while IgG4 creates immune complexes with dietary antigens (notably cow’s milk proteins). IgG4 levels correlate with histologic severity, but do not act as the primary cause of disease [91–93].

## Treatment Approaches for Eosinophilic Esophagitis

Untreated EoE causes chronic inflammation, leading to esophageal fibrotic remodeling and stricture formation, functional impairment, and a significant decline in patients’ health-related quality of life [94]. The therapeutic goal of EoE treatment is to relieve patient symptoms and prevent long-term complications by achieving histologic remission [95]. Current EoE treatment options include eliminating allergenic foods from the diet or using pharmacologic therapy such as proton pump inhibitors, swallowed corticosteroids, or dupilumab. In this discussion, we will review the mechanisms of currently available therapies for eosinophilic esophagitis (EoE), focusing on how they target underlying disease pathways to reduce inflammation and improve symptoms. We will then explore therapies that have been or are currently under investigation, highlighting the pathologic mechanisms targeted by potential treatment strategies.

### Current Clinical Therapies

#### Dietary Management

Dietary therapy is the primary non-pharmacologic approach for eosinophilic esophagitis, focusing on removing food allergens that trigger the condition to achieve symptom relief and histological remission. Various strategies have been studied, including elemental diets, allergy test-guided food restrictions, and empirical elimination diets. These methods directly target the cause of EoE by removing the offending antigens from the diet; however, there are several potential limitations. These include higher costs for food substitutions, social impacts from food avoidance, the possible need for more frequent endoscopies, and nutritional risks.

Elemental diets primarily depend on amino-acid based formulas to eliminate all antigens from the diet and are highly effective in inducing histological remission and quickly relieving symptoms [96, 97]. Despite their effectiveness, elemental diets are very restrictive and typically used only in specific clinical situations, often requiring the guidance of a clinical nutritionist due to their restrictive nature. Allergy test-guided elimination diets have been shown to have poor correlation with EoE-triggering foods as confirmed by biopsy [98, 99], and are no longer recommended. Empiric food elimination diets (FEDs) involve removing the most common food allergens that cause EoE and have been extensively studied because they are the most practical approach for many patients [95]. Randomized controlled trials demonstrated that histological remission rates with a one-food elimination diet (1FED; milk) were similar to those achieved with a four-food elimination diet (4FED; milk, egg, wheat, soy) in children and a six-food elimination diet (6FED; milk, wheat, egg, soy, fish/shellfish, nuts) in adults, supporting 1FED as a suitable initial dietary therapy for EoE [100, 101].

Although the immunologic mechanisms of EoE are not fully understood, the overall success of dietary elimination highlights that delayed cell-mediated hypersensitivity is a key part of the disease’s development. Esophageal biopsy tissue from EoE patients shows a clonally expanded pathogenic Th2 population that expresses the esophageal homing receptor *GPR15* [60]. Patients with EoE who respond to a milk elimination diet have a higher frequency of circulating milk-antigen-specific Th2 cells that produce greater levels of type 2 cytokines when stimulated ex vivo with milk allergens.

#### Proton Pump Inhibitors (PPIs)

Proton pump inhibitors (PPIs) are cost-effective, widely available, and generally tolerated as a first-line pharmacological treatment option for EoE [95]. Over time, the role of PPIs has shifted from a diagnostic tool used to exclude proton-pump inhibitor-responsive esophageal eosinophilia to a first-line anti-inflammatory treatment for EoE [102].

A systematic review and meta-analysis of 33 studies found that high-dose PPI therapy achieved histological remission and symptomatic improvement in 50.5% and 60.8% of patients, respectively [103]. A recent study of 7304 patients reported a clinical response in 65% and histological remission in 45.4% of patients, with no significant differences between children and adults [104]. Deep histologic remission, defined as < 5 eos/hpf, was observed in 34.4% of patients, with no difference between pediatric and adult populations [104].

Multiple mechanisms have been proposed to explain the effect of PPIs in EoE. These are thought to be independent from the known role of PPI as an acid suppressant, and include reducing *STAT6-*mediated gene expression, blocking eosinophil migration into the esophagus, inhibiting *ATP12A*, and activating the aryl hydrocarbon receptor [105]. Research has shown that PPIs decrease STAT6 phosphorylation, which in turn reduces the expression of eotaxin-3 and other epithelial chemokines [106, 107]. PPI therapy has been shown to restore gene expression in biopsy tissue and the esophageal epithelium, reversing the allergic inflammatory gene expression profile characteristic of EoE [108]. This has been linked to improved epithelial barrier function *in vitro.*

Although it’s unclear how PPI decreases STAT6 phosphorylation, studies have indicated that downstream signaling, such as eotaxin-3 gene expression, relies on calcium flux through P-type H+, K+-ATPase channels like *ATP12A* in epithelial cells [109]. This suggests that some effects may be mediated by non-gastric proton pump expression within the epithelium. Single-nucleotide polymorphisms in *CYP2C19*, a main pathway for PPI metabolism, and STAT6 have been shown to affect the therapeutic response to PPI [110]. Additionally, other genes, including ABCB1, which encodes P-glycoprotein involved in PPI absorption, and the ATP4A gene, which encodes the gastric H+/K+-ATPase pump, have a minor role in the effectiveness of PPIs [111].

#### Swallowed Topical Corticosteroids (STCs)

STCs have proven effective in treating EoE. Multiple clinical trials have consistently shown that STCs can induce histological remission rates of 62% to 86% across different studies and also improve clinical symptoms and endoscopic appearance compared to placebo [112, 113]. All initial trials of budesonide and fluticasone included off-label use of inhaled topical corticosteroid preparations modified for oral use in EoE. The US FDA has approved a budesonide oral suspension for treating EoE patients 11 years and older, and budesonide orodispersible tablets have been approved in Europe, Australia, and Canada. Other formulations, like mometasone delivered via the EsoCap system, are also under investigation [114].

Swallowed corticosteroids like fluticasone and budesonide are attractive treatment options for EoE because they work locally on the esophageal lining while reducing systemic effects. Both medications undergo significant first-pass metabolism in the liver after oral intake, resulting in very low systemic bioavailability and restricting their effects mainly to the esophagus. These factors contribute to the generally favorable side effect profile of STCs. The most common side effect of STCs is esophageal candidiasis (3.8%−23.7%) [115]. Rare complications include adrenal suppression, bone demineralization, and slowed growth, with the latter most often seen in younger children [116].

Within the esophagus, corticosteroids bind to intracellular glucocorticoid receptors (GRs), and this complex translocates to the nucleus, where it attaches to glucocorticoid response elements (GREs) in DNA to regulate gene transcription. Corticosteroids increase the production of anti-inflammatory mediators (e.g., IL-10, annexin-1) while inhibiting the transcription of pro-inflammatory genes driven by transcription factors such as NF-κB and AP-1. Consequently, there is a reduction in the production of type 2 cytokines (IL-5, IL-13), eotaxin-3, and other chemokines that recruit and activate eosinophils. Corticosteroids have broad effects on various cells in the esophageal mucosa, decreasing mast cell activity, antigen presentation by dendritic cells, and epithelial proliferation, which leads to reduced tissue remodeling and fibrosis [74, 117, 118].

#### Anti-IL4Rα Monoclonal Antibody Therapy

Dupilumab is a humanized monoclonal antibody that targets the alpha subunit of the IL-4 receptor (IL-4Rα). In doing so, it blocks signaling through both IL-4 and IL-13 receptors [119]. The IL-4Rα subunit can combine with the γc chain to form IL-4R type I, which binds IL-4, or with IL-13Rα1 to form IL-4R type II, which binds both IL-4 and IL-13. Both of these are crucial for atopic diseases [120]. Dupilumab is approved for treatment of EoE in patients at least 1 year of age and weighing 15 kg or more in the US, Canada, and EU [121, 122].

Clinical trials have demonstrated that dupilumab significantly improves clinical symptoms, endoscopic scores, and histologic inflammation in patients with EoE [122]. Two phase 3 studies found that 59–60% of patients receiving weekly dupilumab achieved histologic remission (≤ 6 eos/hpf) after 24 weeks, compared to only 5–6% in the placebo group [112, 123]. Weekly dupilumab was linked to notable reductions in dysphagia, and long-term data showed that 56% of patients on dupilumab maintained histologic remission for up to 52 weeks, with 60% achieving remission after switching from placebo to weekly dupilumab [112]. A phase 3 trial in children aged 1–11 years (EoE KIDS) found that dupilumab induced histologic remission in 68% of patients on the higher-exposure regimen and 58% on the lower-exposure regimen, compared to 3% with placebo at 16 weeks. These benefits were sustained through 52 weeks, and the safety profile was consistent with that observed in older age groups [124]. Common adverse events reported in trials included injection site reaction/erythema, nasopharyngitis, and headache [112, 122, 125]. Currently, dupilumab is recommended for patients who do not respond to or cannot tolerate PPIs or STCs, those with multiple atopic comorbidities (such as atopic dermatitis, asthma, or chronic rhinosinusitis with nasal polyposis), or patients with more severe disease [121, 126]. Further research is necessary to clarify dupilumab’s role in newly diagnosed patients, as well as its long-term efficacy and safety.

#### Limitations in Current Therapy

The underlying pathophysiology of EoE is complex, and no single treatment has proven to be more effective than others in clinical trials. There are no diagnostic tests that predict response to treatment in EoE, and the decision on which therapy to start is a shared choice between the patient and clinicians, based on patient preferences and values. Each of the current treatment options carries a significant risk of therapeutic nonresponse, resulting in the failure to relieve symptoms and achieve histologic remission. It is estimated that 32–58% of patients will not respond to their first-line treatment for EoE [127]. Additionally, some patients do not respond to any of the available medications as monotherapy and require combinations of therapies to achieve remission. However, the risks, benefits, and optimal forms of combination therapy in EoE are not well understood. Therefore, there is a continued need for new treatments for EoE to address these treatment gaps.

### Insights from prior clinical studies

#### Anti-IL5 Monoclonal Antibody Therapy

IL-5 plays a vital role in the proliferation, maturation, activation, and survival of eosinophils [128]. Given IL-5’s central role in eosinophil recruitment, activation, and survival, therapies targeting this pathway have been examined as potential treatments for eosinophilic esophagitis. In multiple clinical trials, agents such as mepolizumab, reslizumab, and benralizumab consistently lowered esophageal eosinophil counts, but improvements in patient-reported symptoms like dysphagia were limited or absent.

Mepolizumab showed histologic benefits in early trials, with reductions in esophageal eosinophilia and markers of tissue remodeling, including tenascin C, TGF-β1, and eosinophil-derived neurotoxin deposition. However, these changes did not lead to significant symptom improvement, and ongoing epithelial abnormalities such as basal zone hyperplasia and spongiosis were still observed [129, 130]. Reslizumab also lowered eosinophil counts in pediatric studies without immediate symptom relief, although long-term follow-up indicated modest improvements in both histology and clinical outcomes [131, 132]. Benralizumab, which causes eosinophil depletion via antibody-dependent cytotoxicity, achieved high rates of histologic remission but did not relieve dysphagia symptoms [133, 134].

These trials underscored the need for validated tools to measure symptomatic response to treatment and highlighted that eosinophils are not the only mediators of clinical symptoms in EoE. Specifically, the broader roles of immune cells (such as mast cells, basophils, and T lymphocytes), epithelial cells, and fibroblasts are minimally affected by IL-5 therapy.

#### Anti-siglec-8 Monoclonal Antibody Therapy

Lirentelimab is a humanized monoclonal antibody that targets Siglec-8 on eosinophils and mast cells. It inhibits mast cell activity and promotes eosinophil apoptosis [135]. In the KRYPTOS phase 2/3 trial (NCT04322708), lirentelimab improved histologic outcomes in EoE patients but did not achieve the primary endpoint of symptom improvement [136]. An analysis revealed a significant histologic response in patients with baseline esophageal eosinophil counts over 24 per high-power field, although the co-primary endpoint of symptom improvement was not met [137]. Analysis ranked monthly lirentelimab 1 mg/kg as most effective for histologic remission in adults and adolescents with active EoE, but it did not show improvements in endoscopy or symptoms [138]. These pivotal trials demonstrated that targeting eosinophils can lead to significant histologic improvement, but eosinophil depletion alone is not enough to produce symptom relief, highlighting the complex, multifactorial nature of EoE beyond eosinophil activity.

#### Anti-KIT Monoclonal Antibody Therapy

Barzolvolimab is a humanized monoclonal antibody that binds the receptor tyrosine kinase KIT, inhibiting its phosphorylation, preventing mast cell activation, degranulation and inhibiting mast cell survival [9, 139]. Barzolvolimab was evaluated in a Phase 2, randomized, double-blind, placebo-controlled Evolve study (NCT05774184) in adults with active EoE. Further development of ant-KIT monoclonal therapy for EoE was halted because no endoscopic or symptomatic improvement was seen at week 12, despite significant depletion of mast cells evident in tissue histology.

#### Anti-IL13 Monoclonal Antibody Therapy

IL-13 contributes to epithelial barrier dysfunction, the induction of eotaxin-3, tissue fibrosis, and basal cell hyperplasia. These pathways are not fully corrected by eosinophil depletion. Anti-IL13 monoclonal antibodies target type-2 inflammatory signaling by binding to IL-13 and blocking its interaction with the receptor.

Two anti-IL13 antibodies have been studied in EoE. Dectrekumab, also known as QAX576, is a human investigational anti-IL13 antibody evaluated in a proof-of-concept trial for its efficacy in EoE. Although there was a significant reduction in tissue eosinophilia, the primary endpoints for histologic response and symptom response were not met [140, 141]. Cendakimab is a humanized monoclonal antibody that blocks IL-13 by inhibiting its binding to both IL-13 receptors (IL-13Rα1 and IL-13Rα2). Although cendakimab is not approved for treatment of EoE, its efficacy has been investigated in several clinical trials. The phase 2 randomized, double-blind, placebo-controlled HEROES study enrolled 99 adults with active EoE, who received weekly subcutaneous doses of either 180 mg or 360 mg of cendakimab or placebo for 16 weeks, followed by an optional open-label extension. Cendakimab significantly reduced esophageal eosinophil counts, histologic and endoscopic scores, and disease severity compared with placebo [142]. In the 52-week extension, these improvements were maintained with higher symptom remission rates, including in patients initially on placebo or non-responders [143]. In the multicenter, multinational, randomized, double-blind, placebo-controlled induction and maintenance phase 3 trial (NCT04753697), cendakimab significantly improved dysphagia symptoms and achieved histologic remission compared with placebo at 24 weeks, with these benefits maintained through 48 weeks, and was generally safe and well tolerated [144].

Overall, the success of cendakimab and dupilumab (anti-IL4Rα) demonstrates the importance of type 2 signaling in EoE development and highlights that effective treatment for EoE will likely need to target multiple immune pathways and effector cells beyond eosinophils to address both inflammatory and structural aspects of the disease.

#### Anti-TSLP Monoclonal Antibody Therapy

Tezepelumab is a fully human anti-TSLP antibody being investigated as a potential treatment for EoE. It works by targeting circulating TSLP and preventing its binding to the TSLP receptor [145]. It has received Orphan Drug designation for EoE from FDA [146]. A case report described an adolescent with active EoE who, while receiving tezepelumab for asthma, achieved sustained histologic and endoscopic remission, normalization of the EoE transcriptome, resolution of dysphagia, and tolerated monthly injections for over two years without adverse effects [147]. A phase 3 clinical trial (CROSSING study, NCT05583227) is evaluating the efficacy and safety of Tezepelumab in 360 adolescents and adults with EoE, with primary endpoints of histologic response (≤ 6 eos/hpf) and change in Dysphagia Symptom Questionnaire (DSQ) score at week 24 [148]. Tezepelumab’s effectiveness in asthma appears to be independent of eosinophil levels. TSLP is considered a promising approach because TSLP is an epithelial alarmin that initiates the inflammatory process in EoE [149].

#### Anti-IgE Monoclonal Antibody Therapy

Omalizumab, a humanized monoclonal anti-IgE antibody, inhibits mast cell activity by blocking the binding of free IgE to its receptor. While an early open-label study reported histologic remission in 33% of patients, subsequent randomized controlled trials did not demonstrate any significant benefit in symptoms or histologic activity compared to placebo [150, 151], highlighting that IgE and mast cells may play a role, but are not central to EoE pathogenesis. Omalizumab is not recommended as an EoE treatment [152].

#### Leukotriene Receptor Antagonists

Montelukast is a competitive, selective leukotriene receptor antagonist [153]. Despite its role in other allergic conditions, such as asthma, montelukast has not shown efficacy in inducing or maintaining remission in EoE. The use of montelukast or other anti-leukotriene agents is not recommended for EoE treatment [153, 154].

#### Anti-TNFα Monoclonal Antibody Therapy

Infliximab is a chimeric monoclonal antibody that inhibits TNFα in both its soluble and membrane-bound forms [155]. A pilot trial of infliximab in three adults with severe, corticosteroid-dependent EoE found that, although well tolerated, the treatment did not improve tissue eosinophilia or EoE-related symptoms, indicating that targeting TNF-α alone is not sufficient to control disease activity [155]. Retrospective case series have linked the initiation of infliximab for inflammatory bowel disease with the development of peripheral eosinophilia and eosinophilic esophagitis [156]. Infliximab is not recommended for EoE treatment [154].

### Novel therapeutics

#### Integrin Antagonists

The α4β7 integrin plays a crucial role in directing leukocytes to the gastrointestinal tract by binding to the mucosal vascular addressin cell adhesion molecule 1 (MAdCAM-1) receptor on endothelial cells [152]. Vedolizumab, an anti-α4β7 monoclonal antibody therapy, is approved to treat inflammatory bowel disease (IBD). In case reports involving two patients receiving treatment for Crohn’s disease, vedolizumab led to histologic remission of EoE [154, 157]. Natalizumab is a humanized monoclonal antibody that broadly inhibits integrin-mediated leukocyte migration by blocking the α4 subunit of both α4β1 and α4β7 integrins and is approved in the United States for treating adults with relapsing multiple sclerosis or moderate-to-severe Crohn’s disease [158]. Treatment of EoE using natalizumab in a female patient for multiple sclerosis has been reported to induce both clinical and histological remission of eosinophilic esophagitis for up to three years. This patient had previously shown disease refractory to PPIs, STCs, and a six-food elimination diet [158]. The side-effect profile of natalizumab includes increased risk of infections, including progressive multifocal leukoencephalopathy, an opportunistic infection caused by the JC virus. There have been no clinical trials testing integrin antagonist therapy in EoE. Considering the increasing number of patients with inflammatory bowel disease who also exhibit manifestations of EoE, further exploration of this approach could be beneficial, as it might target both disease processes.

#### Potassium Competitive Acid Blockers (P-CABs)

P-CABs are a newer class of antisecretory drugs that may be effective for EoE. P-CABs reversibly inhibit the potassium channel of the H⁺/K⁺-ATPase proton pump, unlike proton pump inhibitors that bind irreversibly [159]. Vonoprazan and rebaprazan are marketed in Japan and South Korea, respectively, with ongoing research to confirm their efficacy and safety in Western populations [95]. In a retrospective review of 114 Japanese patients, there was no significant difference in the symptomatic (72.7% vs. 64.2%, *p* = 0.367), endoscopic (48.5% vs. 39.5%, *p* = 0.502), and histologic response rates (39.4% vs. 25.9%, *p* = 0.274) between patients who received vonoprazan and rabeprazole [160]. A case series of EoE patients unresponsive to 12 weeks of esomeprazole found that switching to vonoprazan resolved symptoms and histology in 3 of 4 cases [161].

#### Anti-IL-15 Monoclonal Antibody Therapy

CALY-002 is a humanized monoclonal antibody that targets both free IL-15 and IL-15 bound to IL-15Rα [9, 162]. IL-15 is considered a potential therapeutic target due to its expression in the gastrointestinal epithelium and its regulatory role in the gut mucosa [163]. Early results from an open-label cohort study suggest that CALY-002 is well-tolerated and shows promise in reversing esophageal inflammation (including eosinophilia and tissue damage) and improving symptoms in patients with EoE, especially those who have not responded to corticosteroids [162]. CALY-002 is currently in Phase 1 trials for celiac disease.

#### JAK-STAT Inhibitors (Tofacitinib)

 In vitro inhibition of the STAT6 pathway using agents like the JAK inhibitor ruxolitinib, the STAT6 inhibitor AS1517499, and the pyrimidine synthase inhibitor leflunomide has shown the ability to block multiple IL-13-induced pathological changes in epithelial and fibroblast function [164]. Targeting the JAK-STAT pathway also broadly influences immune function, as JAK-STAT signaling is involved in many cytokine pathways beyond type 2 signaling. Tofacitinib, a pan-JAK inhibitor, has shown preliminary efficacy in other eosinophilic conditions, including hyper-eosinophilic syndrome, bronchial asthma, and eosinophilic fasciitis. Tofacitinib was reported to induce clinical and endoscopic remission in a patient with refractory EoE [165]. However, JAK inhibitors have been linked to modest increases in serious systemic side effects, including a higher risk of infection, thromboembolism, and nonmelanoma skin cancer [166]. Further studies are needed to determine whether JAK inhibitors could benefit some patients with difficult-to-treat EoE or EoE associated with lower tract eosinophilic gastrointestinal disease.

#### Sphingosine 1-Phosphate (S1P) Receptor Modulators (Etrasimod)

Etrasimod is a selective S1P receptor modulator (targeting S1PR1, S1PR4, and S1PR5) and presents a promising therapeutic approach for EoE by specifically influencing immune cell infiltration [167]. In the Phase 2 VOYAGE study (NCT04682639) of adults with active eosinophilic esophagitis, estrasimod 2 mg once daily significantly reduced esophageal peak eosinophil counts, improved endoscopic and histologic features, and alleviated symptoms including dysphagia, while demonstrating a favorable safety profile up to 24 weeks [167]. These findings support further evaluation of etrasimod in EoE.

#### CRTH2 Antagonists (OC000459/Timapiprant)

The CRTH2 receptor is found on Th2 cells, ILC2 cells, eosinophils, and basophils, where it mediates their activation and chemotaxis [168]. In a Phase 2 randomized, double-blind, placebo-controlled trial involving 26 corticosteroid-dependent or -refractory adults with EoE, treatment with OC000459 for 8 weeks significantly lowered esophageal eosinophil counts and inflammatory biomarkers compared to placebo. However, it did not lead to meaningful improvements in patient-reported symptoms or endoscopic features, which ultimately limited its further development for EoE [116].

#### Peroxisome proliferator-activated receptor-γ Agonists (Thiazolidinediones)

Peroxisome proliferator-activated receptor-γ *(*PPAR-γ) is upregulated in eosinophilic esophagitis (EoE), exhibiting increased expression in CD4⁺ T cells and fibroblasts (enhanced by interleukin-4), and is detectable within the epithelium and lamina propria of active EoE, while being absent in healthy esophageal tissue [114]. Thiazolidinediones (TZDs), including rosiglitazone and pioglitazone, constitute a class of anti-hyperglycemic medications that activate PPAR-γ and have been suggested as promising candidates for targeting fibrosis in EoE. These agents have been shown to suppress TGF-β1-induced myofibroblast differentiation, reduce fibrotic gene and protein expression, inhibit p38 phosphorylation, and demonstrate greater potency than budesonide in decreasing collagen-1α1 expression in EoE fibroblasts [169]. The results associated with TZDs indicate their potential to address the fibrotic complications of EoE, thereby offering a promising direction for future targeted therapeutic strategies aimed at specific tissue repair mechanisms.

#### Angiotensin II Receptor Blockers (ARBs)

Losartan is an ARB commonly used to treat high blood pressure, has also been shown to inhibit angiotensin-II mediated TGF- β signaling [74], leading to anti-fibrotic effects [170]. This suggests that it could be a potential option for preventing or reversing fibrotic complications in EoE. An open-label trial (NCT01808196) and a Phase 2 trial (NCT03029091) have been completed, though published results are still limited. Early data from these studies report modest effects of losartan on eosinophilic infiltration, histologic remission, endoscopic scores, and symptoms, with no available data on fibrosis or esophageal compliance.

#### Epithelial Barrier Protection

Given the importance of epithelial barrier integrity in EoE, there is interest strategies to improve the epithelial barrier. Sucralfate, an aluminum salt of sucrose sulfate and a cytoprotective agent, is being studied as a potential EoE treatment [171]. A Phase I trial (NCT0235307) was completed but no results are reported yet. HIF-1α stabilizers like the pan-hydroxylase inhibitor DMOG can restore barrier molecule expression on esophageal biopsies [172], suggesting targeting HIF-1α may help restore barrier integrity. However, the lack of selective compounds and systemic side effects pose challenges, requiring more research. In vitro, SCFAs like butyrate and propionate suppress CAPN14 and boost FLG and junctional proteins, strengthening the barrier [173]. Zemaira, an alpha-1 trypsin inhibitor, inhibits kallikrein-5 (KLK5) activity by antagonizing protease-activated receptor P2, increasing SPINK7 expression [45, 174]. Currently, a Phase II trial (NCT05485155) is investigating whether Zemaira accumulates in the esophagus to reduce protease activity and improve the epithelial barrier in EoE.

## Conclusion and Future Perspectives

Therapeutic trials in eosinophilic esophagitis have provided valuable insights into disease mechanisms, emphasizing that eosinophils, while key to diagnosis, are not the only drivers of symptoms or tissue changes. Studies involving dietary elimination, proton pump inhibitors, corticosteroids, and biologics targeting IL-5, IL-13, and other pathways collectively demonstrate the multifaceted nature of EoE’s underlying processes, including epithelial barrier dysfunction, type 2 inflammation, and tissue remodeling. Despite this progress, the current diagnostics still cannot reliably predict how an individual will respond to treatment. This limitation makes personalizing therapy more challenging and underscores the critical need for predictive biomarkers to inform treatment choices.

Equally important, a significant proportion of patients experience no or only partial response to standard therapy, emphasizing the need for ongoing research into new therapeutic targets and combination strategies. The continued development of standardized outcome measures will be crucial for advancing both clinical care and research. These include efforts to establish validated thresholds for symptomatic, histological, and endoscopic remission, improving data comparability and clinical translation [175]. Ultimately, lessons learned from therapeutic studies not only enhance our understanding of EoE pathogenesis but also guide the future toward personalized, mechanism-based treatment approaches that achieve lasting symptom relief, prevent complications, and lessen disease burden.

## Key References

- Dsilva A, Wagner A, Itan M, Rhone N, Avlas S, Gordon Y, et al. Distinct Roles for Thymic Stromal Lymphopoietin (TSLP) and IL-33 in Experimental Eosinophilic Esophagitis. Allergy. 2025.
  Establishes TSLP as an important upstream target in the inflammatory cascade in EoE, and supports the role of anti-TSLP therapy in EoE.
- Kliewer KL, Gonsalves N, Dellon ES, Katzka DA, Abonia JP, Aceves SS, et al. One-food versus six-food elimination diet therapy for the treatment of eosinophilic oesophagitis: a multicentre, randomised, open-label trial. Lancet Gastroenterol Hepatol. 2023;8:408–21.
  This randomized controlled trial of one- versus six-food elimination diet therapy in 129 adult EoE patients demonstrated similar efficacy in inducing remission between both diets, indicating that elimination of milk alone is an acceptable initial therapy choice when compared to the more comprehensive six-food elimination diet.
- Kliewer KL, Abonia JP, Aceves SS, Atkins D, Bonis PA, Capocelli KE, et al. One-food versus 4-food elimination diet for pediatric eosinophilic esophagitis: A multisite randomized trial. J Allergy Clin Immunol. 2025;155:520–32.
  This randomized controlled trial comparing one-food elimination (1FED) versus four-food elimination (4FED) diet therapy in 63 pediatric EoE patients demonstrated modest symptom improvement with 4FED, with no significant differences in endoscopic, histologic, or quality-of-life outcomes.
- Masuda MY, Pyon GC, Luo H, LeSuer WE, Putikova A, Dao A, et al. Epithelial overexpression of IL-33 induces eosinophilic esophagitis dependent on IL-13. J Allergy Clin Immunol. 2024;153:1355–68.
  These studies support the role of IL-33 as an upstream mediator in EoE and show that targeting IL-33 signaling may ameliorate disease.
- Rothenberg ME, Dellon ES, Collins MH, Hirano I, Chehade M, Bredenoord AJ, et al. Efficacy and safety of dupilumab up to 52 weeks in adults and adolescents with eosinophilic oesophagitis (LIBERTY EoE TREET study): a multicentre, double-blind, randomised, placebo-controlled, phase 3 trial. Lancet Gastroenterol Hepatol. 2023;8:990–1004.
  Landmark clinical trial of anti-IL4Ra in EoE patients that demonstrated direct inhibition of type 2 cytokine signaling significantly improved symptoms and histology.
- Sharma M, Leung D, Momenilandi M, Jones LCW, Pacillo L, James AE, et al. Human germline heterozygous gain-of-function *STAT6* variants cause severe allergic disease. Journal of Experimental Medicine. 2023;220:e20221755.
  This study identified heterozygous GOF variants in STAT6 as a novel autosomal dominant allergic disorder that included a profound phenotype, including severe atopic dermatitis, hypereosinophilia with eosinophilic gastrointestinal disease, asthma, elevated serum IgE, IgE-mediated food allergies, and anaphylaxis.

## Data Availability

No datasets were generated or analysed during the current study.
